# The Cytology, Isozyme, HPLC Fingerprint, and Interspecific Hybridization Studies of Genus *Epimedium* (Berberidaceae)

**DOI:** 10.1155/2013/271578

**Published:** 2013-11-19

**Authors:** Lin-Jiao Wang, Mao-Yin Sheng

**Affiliations:** ^1^School of Vocation and Technology, Guizhou Normal University, Guiyang, Guizhou 550001, China; ^2^Institute of South China Karst, Guizhou Normal University, Guiyang 550001, China; ^3^State Key Laboratory Incubation Base for Karst Mountain Ecology Environment of Guizhou Province, Guiyang 550001, China; ^4^School of Life Science, Nanjing University, Nanjing, Jiangsu 210093, China

## Abstract

104 samples from 27 accessions belonging to 12 species of genus *Epimedium* were studied on the basis of cytology observation, POD (i.e., peroxide) isozyme, high performance liquid chromatography (i.e., HPLC) fingerprint, and interspecific hybridization. The cytology observation showed karyotypes of twelve species studied; all are 2A symmetry type of Stebbins standard and similar to each other, and except for karyotype of *E. leptorrhizum* which is 2n = 2x = 8m (2SAT) + 4sm, the rest are 2n = 2x = 6m (2SAT) + 6sm. Chromosomes C-banding of barrenwort species varies, with 15 to 22 bands, consisting of centromeric bands, intercalary bands, terminal bands, and middle satellite bands. Results of POD isozyme showed that the zymographs vary greatly and sixteen bands were detected in the eleven species, and each species has its own characteristic bands different from the others. Studies on the HPLC fingerprint showed that the HPLC fingerprint of different species has characteristic peaks, divided into two regions (retention time < 10 min and retention time > 10 min). Results of interspecific hybridization showed that crosses of any combination among seven species studied are successful and the rates of grain set vary greatly. Based on these results, the system and phylogeny of this genus were inferred.

## 1. Introduction

Barrenwort (*Epimedium*, Berberidaceae) is a traditional Chinese medicine named as Yinyanghuo, Xianlinpi, and Yangheye in China [1–3]. The plant is effective in strengthening kidneys and curing rheumatism, widely used in the treatment of osteoporosis, hypertension, coronary heart disease [4], caducity, cancer, and so on [1–3]. Many flavonol glycosides have been isolated from a number of *Epimedium* plants, though the relation between these flavonol glycosides and the pharmacological activities of the drug is still obscure [4]. In Japan, Europe, and America, barrenwort are also very popular as graceful garden plants.

*Epimedium* is a genus of the Old World. Members disperse from Japan to Algeria and mainly occur in eastern Asia and the Mediterranean lands [5]. Approximately 80% of the total species are found in central-southeastern China [6]. Linnaeus recorded this genus and its type species *E. alpinum *in 1753. After that, Morren and Decaisne (1834); Franchet (1886); Komarov (1908); and Stearn (1938, 2002) made monographic and systematic study of *Epimedium*, respectively. Up till now, more than 60 species of *Epimedium* are recognized. Stearn [5, 7] established the most comprehensive classification system of this genus. In his monograph, he arranged the genus into two subgenera,* Epimedium* L. and *Rhizophyllum* (Fischer & Meyer) Stearn, mainly based on whether the flowering stem has leaves or not, and he divided the subgenus, *Epimedium*, into four sections mainly according to their geographical distribution and infrageneric relationship. Among the four sections, members of section *Diphyllon* (Kom.) Stearn were all endemic to China and subdivided into four series: *Campanulatae* Stearn, *Davidianae* Stearn, *Dolichocerae* Stearn, and *Brachycerae* Stearn based on corolla characteristics such as petal type, the form and relative size of the inner sepals and petals, and flower dimension.

Up to now, lots of works have been adopted to deduce the system and evolution of this genus such as pollen morphology [8, 9], cytology [10, 11], isozyme [12], molecular biology [13–16], biogeography [6, 17], chemical classification [4, 12, 18], and so on. Results showed that the infrageneric classification was generally consistent with its phytogeographic distribution, and the phylogenetic relationship among sections was basically clear in *Epimedium*. However, for lots of *Epimedium* species with multifarious petals are nested together in China and the petal evolved is quite controversial, the system of Chinese species is still not revealed well [18]. Recently, many further researches were performed for the system and phylogeny of *Epimedium *[17, 19, 20]. As an intractable genus in taxonomy, however, there still remain several problems about the classification and phylogenetic relationship of *Epimedium* species, especially for the Chinese *Epimedium* species. As a result, it is essential to establish a natural arrangement of *Epimedium*.

 In this paper, 104 samples from 27 accessions belonging to twelve barrenwort species were systemically studied on the cytology, peroxide (POD) isozyme, high performance liquid chromatography (HPLC) fingerprint, and interspecific hybridization to infer the system and phylogeny of this genus.

## 2. Materials and Methods

### 2.1. Plant Materials

We collected 104 samples from 27 accessions belonging to twelve barrenwort species (Table 1) and planted them in pots filled with compost (one plant per pot). All the pots were kept in greenhouses at the Institute of Plant Genetics and Breeding (IPGB) under identical conditions. The entire study was carried out at the same institute.

### 2.2. Karyotype Analyses

Somatic chromosomes were studied in root meristems of individuals which were pretreated in *α*-bromine naphthalin at 23°C for 5 h, then fixed in ethanol : acetic acid (3 : 1) for 4~12 h, and stored in 70% aqueous ethanol. Root tips were stained according to Feulgen technique, meristems were macerated in 1 mol/L hydrochloric acid at 60°C for 10 minutes before squashing, and slides were made permanent using Balata as the mounting medium. At least 10 metaphases were drawn for each population using an Olympus BX51 camera (Olympus, Japan), selecting the five best for measurements. The nomenclature used for the description of the chromosome morphology is that proposed by Levan et al. [21].

For the numerical characterization of the karyotypes, the following parameters were calculated: (1) total chromosome length of the haploid complement (TCL); (2) mean chromosome length (CL); (3) mean centromere index (CI); (4) intrachromosomal asymmetry index (*A*_1_) = 1 − [∑(*b*/*B*)/*n*]; and (5) interchromosomal asymmetry index (*A*_2_) = *s*/*x*, where *b* and *B* are the mean length of short and long arms of each pair of homologues, respectively, *n* is the number of homologues, *s* is the standard deviation, and *x* is the mean chromosome length. Karyotype asymmetry has been determined using the categories of Stebbins [22].

### 2.3. Giemsa C-Band Analyses

Somatic chromosomes were studied in root meristems of individuals which were pretreated in 0.2% aqueous colchicine at 23°C for 4 h, then fixed in ethanol : acetic acid (3 : 1) for 24 h, and stored in 70% aqueous ethanol; meristems were squashed in 45% acetum. Cover glasses were uncovered after freezing, and then samples were set in air at 23°C for five days before being treated by Giemsa chromosome banding. The improved BSG method of chromosome C-banding was used. Mature samples were treated in 0.2 mol/L hydrochloric acid for 50 minutes, treated in 50% aqueous Ba(OH)_2_ at 60°C for 30 minutes after being washed by water, then treated in 2 × SSC solution at 60°C for 5 h after being washed by water, and finally dyed in 3% aqueous Giemsa (pH 6.8) for 1 h. Chromosomes C-bandings among species were compared by the method of Li [23]. 

### 2.4. PAGE Analyses of POD Isozyme

The polyacrylamide gel electrophoresis (PAGE) method of POD isozyme analyses was described by Hu and Wang [24]. The POD was extracted from tender, 2-week-old leaves. The parameters of bio-gel for separation were as follows: *T* (total concentration of Acr and Bis) = 7.5% and *C* (the rate of B is in *T*) = 2.6%. *Rf* value = moving length of the band/moving length of the front line. The coefficient (*c*) and dissimilarity coefficient (*d*) of zymogram were used to analyse the relation among species. *c* = (2*w*/(*a* + *b*)) × 100%, where *a  *= the number of zymogram bands of species A, *b  *= the number of zymogram bands of species B, and *w  *= the number of zymogram bands common to species A and B, and *d* = 100% − *c*. Idiograms were drawn based on the results and analyzed.

### 2.5. HPLC Fingerprint of Flavonoids

The HPLC fingerprints were estimated by following the method described by CCP [25]. The HPLC system consisted of an LC2010 and CLASS-VP Ver6.12 computer system (all components from Shimadzu, Kyoto, Japan). A reversed-phase C_18_ (YMC, Japan) column (150 × 4.6 mm) was used for separation. The mobile phase consisted of acetonitrile and water (25 : 75). The flow rate was kept constant at 1.0 mL/min at room temperature (30°C). The detection was performed at 270 nm using an SPD-M10A VP photodiode array detector. The samples delivered into the system, one at a time, measured 10 *μ*L each. All the samples used in the study were from mature leaves.

### 2.6. Interspecific Hybridization

Seven species, *E. myrianthum, E. acuminatum, E. wushanense, E. letorrhizum, E. luodianense, E. simplicifolium, *and* E. yinjiangense* grown in green house, were selected as female and male parents, crossed with each other. As flowers opened (before anther dehiscence), the species chosen as female parents were emasculated and pollinated by rubbing them with newly dehisced anthers of the male parents. More than 200 flowers were pollinated per cross. Remaining buds in the inflorescences were removed. Twenty days later, the rate of grain set (%) = (the number of grain set/the number of pollinated flowers) × 100. Based on the rate of grain set, the relations of these seven species were compared by the hierarchical cluster of SPSS [26].

## 3. Results

### 3.1. Morphological Observation and Karyotype Analyses of Chromosomes

Based on the size of flower, twelve species can be grouped into two types, type one is small flower including *E. sagittatum *and *E. myrianthum, *type two is large flower, the rest. The results of chromosomal observations of twelve species showed all species studied were diploid (2n = 2x = 12) and had one pair of middle satellite chromosomes. Expect for the middle satellite chromosomes of *E. leptorrhizum*, *E. simplicifolium *and *E. wushanense* are in short arms of no. 2 chromosomes; the rest are no. 1 chromosomes. Tetraploid cells sporadically were found in *E. letorrhizum* root meristems among twelve species. The total length of haploid complement (TCL) of *E. yinjiangense *and *E. alpinum* are obviously small, 29.23 ± 2.36 *μ*m and 32.96 ± 1.34 *μ*m, respectively. And the range of chromosome length of *E. yinjiangense* is 2.15~2.92 *μ*m and *E. alpinum* 2.22~3.06 *μ*m. 

**Figure 1:**
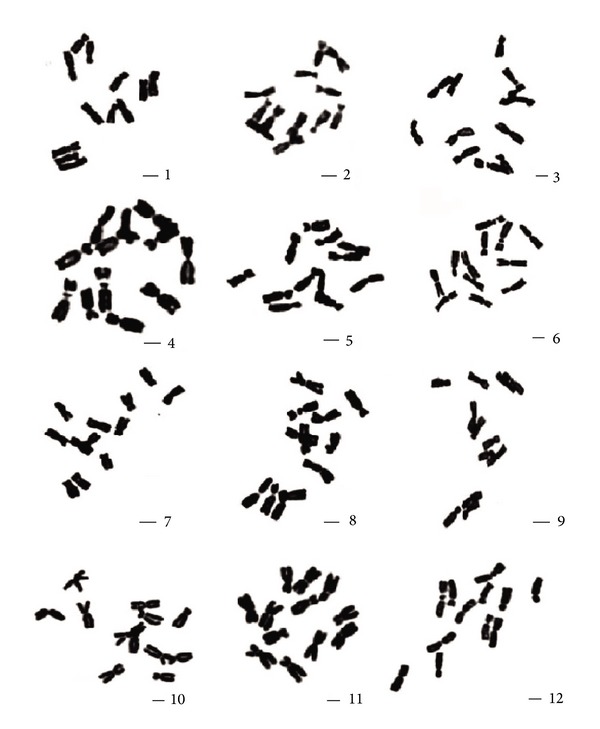
Somatic chromosome of *Epimedium*, all species with 2n = 12. 1–10: large-flower group. 1: *E. acuminatum*. 2: *E. yinjiangense*. 3: *E. luodianense*. 4: *E. leptorrhizum*. 5: *E. simplicifolium*. 6: *E. wushanense*. 7: *E. alpinum*. 8: *E. davidii*. 9: *E. pubigerum*. 10: *E. franchetii*. 11-12: small-flower group. 11: *E. myrianthum*. 12: *E. sagittatum*. Scale bar = 2 *μ*m.

**Figure 2:**
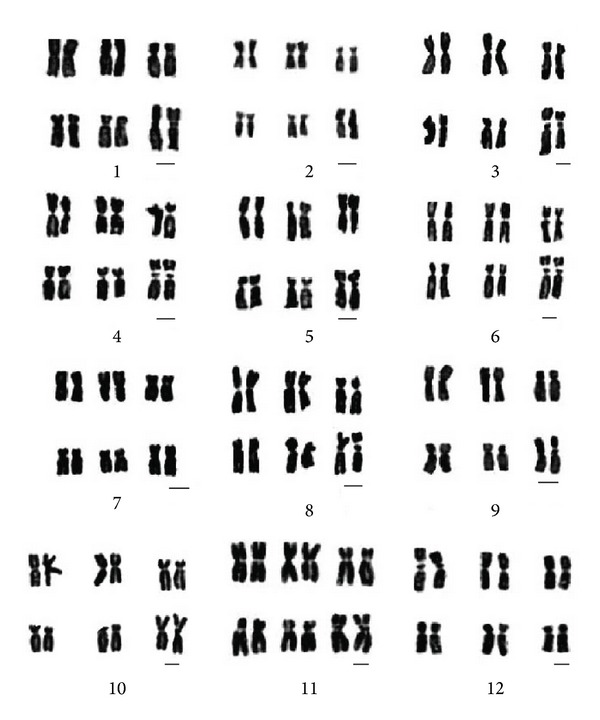
Karyotypes of twelve species metaphase chromosomes, all species with 2n = 12. 1–10: large-flower group. 1: *E. acuminatum*. 2: *E. yinjiangense*. 3: *E. luodianense*. 4: *E. leptorrhizum*. 5: *E. simplicifolium*. 6: *E. wushanense*. 7: *E. alpinum*. 8: *E. davidii.* 9: *E. pubigerum*. 10: *E. franchetii*. 11-12: small-flower group. 11: *E. myrianthum*. 12: *E. sagittatum*. Scale bar = 2 *μ*m.

The parameters (Table 2), figures (Figure 1), and karyotypes (Figure 2) of twelve species metaphase chromosomes were studied. The results showed that their karyotypes are similar to each other, only including m and sm two types chromosome. Twelve species karyotypes are all 2A type of Stebbin's asymmetry categories. Among them, the karyotype formulas of *E. sagittatum*, *E. myrianthum*, *E. acuminatun*, *E. wushanense*, *E. simplicifolium, E. yinjianense*, *E. luodianense*, *E. davidii*, *E. pubigerum*, *E. franchetii,* and* E. alpinum* are 2n = 2x = 6m (2SAT) + 6sm, and *E. leptorrhizum *is 2n = 2x = 8m (2SAT) + 4sm.

### 3.2. Giemsa C-Band Analyses of Chromosomes

**Figure 3:**
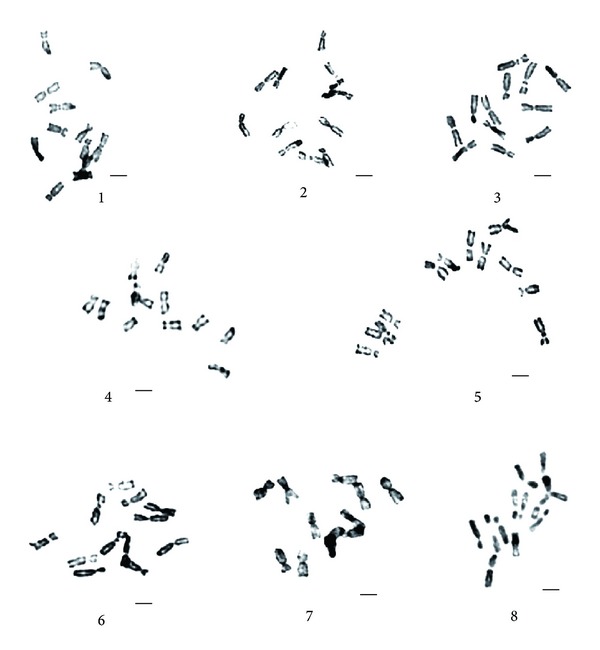
The metaphase chromosomes C, banding in the eight species of genus *Epimedium*. 1: *E. simplicifolium*. 2: *E. leptorrhizum*. 3: *E. wushanense*. 4: *E. alpinum*. 5: *E. davidii*. 6: *E. acuminatum*. 7: *E. pubigerum*. 8: *E. sagittatum*. Scale bar = 2 *μ*m.

**Figure 4:**
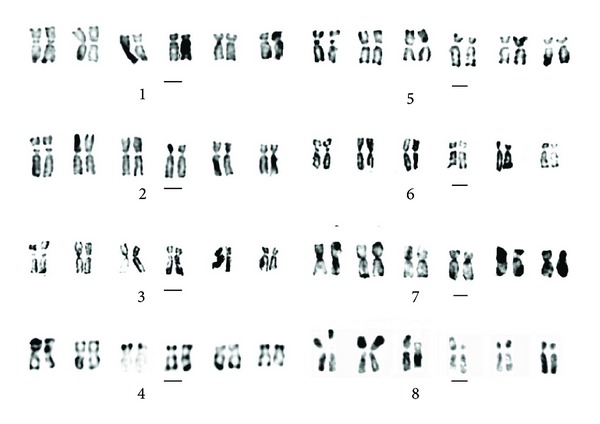
Giemsa C-banded karyotypes in the eight species of genus *Epimedium*. 1: *E. simplicifolium*. 2: *E. leptorrhizum*. 3: *E. wushanense*. 4: *E. alpinum*. 5: *E. davidii*. 6: *E. acuminatum*. 7: *E. pubigerum*. 8: *E. sagittatum*. Scale bar = 2 *μ*m.

**Figure 5:**
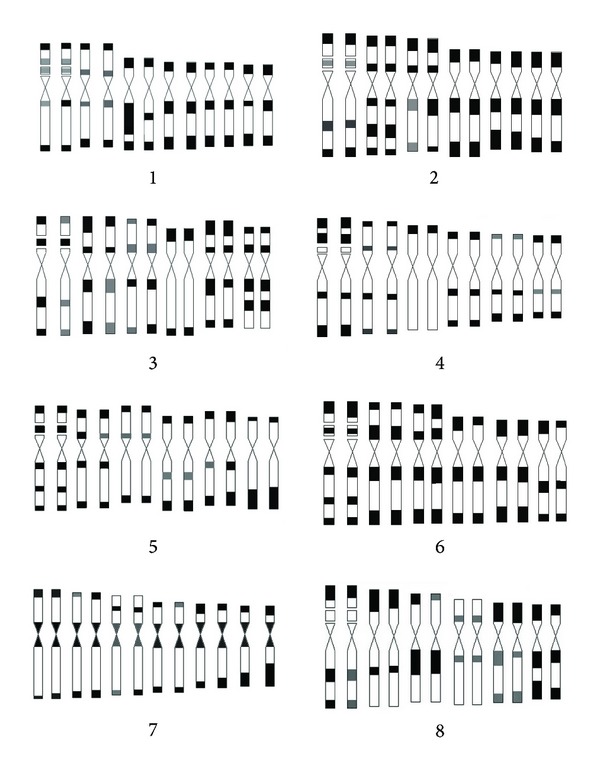
Idiograms of C-banded karyotypes in the eight species of genus *Epimedium*. 1: *E. simplicifolium*. 2: *E. leptorrhizum*. 3: *E. wushanense*. 4: *E. alpinum*. 5: *E. davidii*. 6: *E. acuminatum*. 7: *E. pubigerum*. 8: *E. sagittatum*.

Eight species, *E. sagittatum*, *E. alpinum*, *E. pubigerum*, *E. acuminatum*, *E. simplicifolium*, *E. wushanense*, *E. letorrhizum,* and *E. davidii*, were treated by chromosomes C-banding. The metaphase chromosomes of C-banding, Giemsa C-banding karyotypes and idiograms of C-banding karyotype can be seen in Figures 3, 4, and 5, respectively.

Results showed that the chromosomes C-banding of eight species vary remarkably, with 15 to 22 bands, consisting of centromeric bands, intercalary bands, terminal bands, and middle satellite bands. Based on the number and location of the bands, the tested eight species can be divided into three groups: Group 1 comprises *E. sagittatum*, with only 15 C-bands; Group 2, *E. alpinum and E. pubigerum* native to Germany, with 18 C-bands; Group 3, *E. acuminatum*, *E. simplicifolium*, *E. leptorrhizum,* and *E. davidii*, with the C-band number from 20 to 22.

### 3.3. Peroxide Isozyme

**Figure 6:**
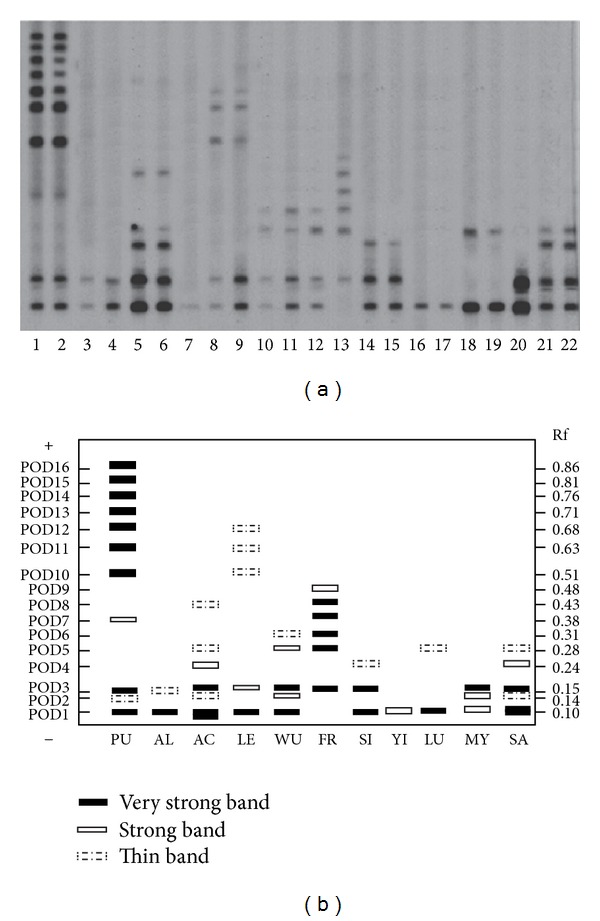
The POD isozyme zymogram and idiogram of twelve species of genus *Epimedium.* 1 = PU1, 2 = PU2, 3 = AL1, 4=AL2, 5 = AC37, 6 = AC22, 7 = LE10, 8 = LE5, 9 = LE6, 10 = WU15, 11 = WU10, 12 = WU16, 13 = FR1, 14 = SI1, 15 = SI2, 16 = YI1, 17 = YI2, 18 = LU1, 19 = LU4, 20 = MY1, 21 = SA1, and 22 = SA2.

The results showed that the POD isozyme zymographs of various leaves of the same accession are similar. For accurate comparison among accessions, the zymographs of all accessions were prepared on a single bio-gel plate (Figure 6). Sixteen bands of POD isozyme (POD 1–16) were detected in the eleven species. Each species has its own characteristic bands, different from others, showing that the POD isozyme zymographs vary greatly among genus* Epimedium*. Among the eleven species, the zymograph bands of *E. pubigerum* were the most abundant, with eleven bands (POD1–3, POD 7, and POD 10–16), *E. yinjiangense* with only one band (POD 1), and *E. luodianense* with two bands (POD 1 and 5). All the zymographs can be divided into three regions, namely, slow (*Rf* ⩽ 0.20), medium (0.2 < *Rf* ⩽ 0.60), and fast (0.6 < *Rf* ⩽ 1.00).

The dissimilarity coefficient (*d*) of isozyme zymograph is the symbol of relationships among species [27]. To find out the relationship of these species, the *d* and $\bar{d}$ value of POD isozyme zymogram were studied and filled in Table 3. The *d* value of *E. acuminatum* and *E. sagittatum* is the lowest, 0.091, and *E. franchetii* and *E. yinjiangense* the highest, 1.00. The $\bar{d}$ values of *E. franchetii*, *E. pubigerum, *and *E. yinjiangense* are higher, respectively, 0.678, 0.669, and 0.621. And the $\bar{d}$ values of *E. sagittatum* and *E. myrianthum* (small-flower), respectively, are 0.432 and 0.418, lower than the others.

### 3.4. HPLC Fingerprint of Flavonoids

**Figure 7:**
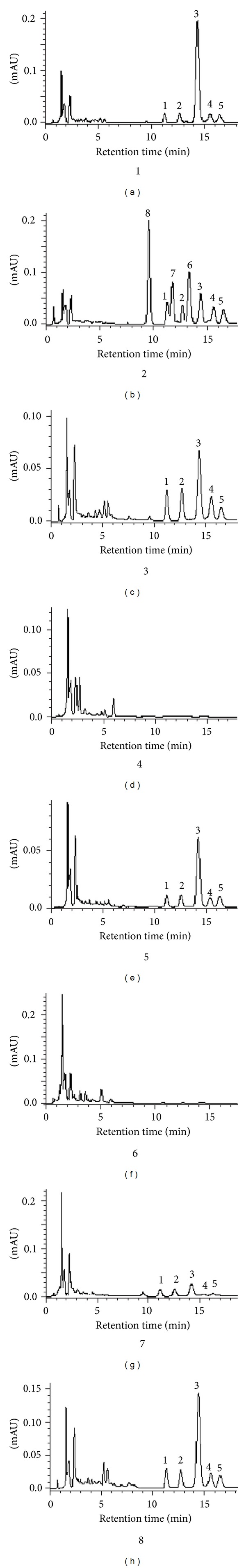
HPLC fingerprint of eight species. 1: *E. wushanense*. 2: *E. sagittatum*. 3: *E. yinjiangense*. 4: *E. leptorrhizum*. 5: *E. simplicifolium*. 6: *E. luodianense*. 7: *E. davidii*. 8: *E. acuminatum*.

The flavonoids HPLC fingerprints of twenty-one barrenwort accessions, belonging to eight species were observed (Figure 7). The result showed that the HPLC fingerprint of different species were different. Each species had characteristic peaks, which were different from those seen in other species. All the HPLC fingerprint peaks can be divided into two regions: Region 1, retention time < 10 min, and Region 2, retention time > 10 min. Based on the number and location of peaks held by the species in Region 2, the eight species can be grouped into three types: Type 1 (*E. sagittatum*) with Peak 1–8, Type 2 (*E. acuminatum*, *E. yinjiangense*, *E. wushanense*, and *E. simplicifolium*) with Peak 1–5, and Type 3 (*E. luodianense* and *E. leptorrhizum*) without any peak, suggesting that the relation among *E. acuminatum*, *E. yinjiangense*, *E. wushanense*, *E. simplicifolium,* and *E. davidii* is near each other; *E. luodianense* and *E. leptorrhizum* are also near. *E. sagittatum* is distant from other species.

### 3.5. Interspecific Crossability

The crossabilities of fourteen accessions belonging to seven species were studied in the aspect of the grain set rate of hybridization (Table 4). Results showed that the hybridizations of any combination among seven species are successful, and the rate of grain set among hybridization combinations vary obviously for the grain set rate of *E. luodianense* and *E. letorrhizum* is the highest (up to 92.44%) and* E. myrianthum* and *E. acuminatum* the lowest (only 15.38%). Based on the rates, seven species studied can be divided into two groups: Group one is only one species, *E. myrianthum*, with lower grain set rate of crossing with other species (almost all under 30%); Group two is the rest, with higher grain set rate (almost all over 30%). 

**Figure 8:**
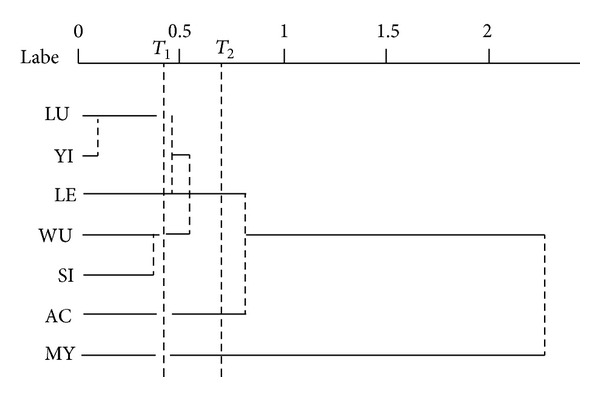
Clustering of seven species by the grain set rate of hybridization.

Results also showed that there are no various differences between the grain set rates of positive and negative hybridization, suggesting that the significance of grain set rats of positive and negative hybridization in symboling the interspecific relationship of this genus is the same. So the means of grain set rats of positive and negative hybridization were analyzed by the unweighted pair-group method (UPGMA) (Figure 8) to find the relationship of seven species researched in this paper. Results showed that *E. myrianthum* is very distant from other six species, and the relation between *E. luodianense* and *E. yinjiangense* is the nearest, and they are nearer related to *E. letorrhizum*.

## 4. Discussion

### 4.1. Generic Evolution

The results showed that karyotypes of twelve species are 2A symmetry type of Stebbins [22] standard and similar to each other only with m and sm chromosomes, and interspecific hybridization easily succeeds, suggesting that *Epimedium* is a very conservative genus in the process of evolution. 

The longer the evolutionary history, the larger the DNA differentiation and the higher the average value of zymogram distance [27]. Su and Liu [28] concluded that *Dysosma *is an age-old genus based on the average value of its isozyme zymogram distance, 0.828. In our study, this value was 0.526, indicating that *Epimedium *may be a more ancient genus in the family Berberidaceae. This result is consistent with those studies results of geobiology [6], pollen morphology [8], molecular biology [13–16], and cytology [23, 29] of this genus, also supported by the deduction about the phylogeny of Berberidaceae by Wu et al.  [2].

Karyotype formulas of *E. sagittatum*, *E. myrianthum*, *E. acuminatum*, *E. wushanense*, *E. simplicifolium*, *E, yinjiangense*, *E. luodianense*, *E. davidii*, *E. pubigerum*, *E. franchetii,* and *E. alpinum* are 2n = 2x = 6m (2SAT) + 6sm, and *E. leptorrhizum* is 2n = 2x = 8m (2SAT) + 4sm. Karyotype formulas of four genus, *Diphylleia*, *Dysasma*, *Podophyllum* and *Sinopodophyllum*, of family Berberidaceae are 2n = 2x = 8m (SAT) + 2st + 2t, 2n = 2x = 8m (2SAT) + 2st (SAT) + 2t, 2n = 2x = 6m (4SAT) + 2sm + 2st + 2t, and 2n = 2x = 8m (2SAT) + 2st (2SAT) + 2t, respectively [23, 29]. Along with the level of evolution advancement, the asymmetry of karyotype will enhance in family Berberidaceae [29]. Obviously, the karyotypes of *Epimedium* are the most symmetrical among genus of Berberidaceae, suggesting that *Epimedium* is the most original genus in the family Berberidaceae.

### 4.2. Interspecific Relationship

Results of chromosomes C-banding, POD isozyme, HPLC fingerprint, and interspecific hybridization researches all show that small-flower species, *E. sagittatum* and *E. myrianthum,* are distant from the ten large flower species, strongly supporting the flower size which is one of the important taxonomy symbols of this genus, and genus *Epimedium* should be classed into two sections: small and large flower.

In large flower species, the relation among *E. leptorrhizum*, *E. luodianense, *and* E. jinjiangense *is near based on results of POD isozyme, HPLC fingerprint, and the grain set rate of interspecific hybridization. Results of HPLC fingerprint that showed peaks in Region 2 of *E. acuminatum*, *E. simplicifolium*, *E. wushanense,* and *E. davidii* are the same with Peaks 1–5, suggesting that the relation of these four species are near, also supported by the result of interspecific hybridization research. Results of chromosomes C-banding showed that *E. alpinum *and* E. pubigerum* were divided into the same group, suggesting that the relation of these two species are near. Results of POD isozyme analyses showed that the zymograph of *E. franchetii* varies greatly from the others, suggesting that *E. franchetii *is distant from the rest.

Modernistic taxonomy sparkplug that species set by classical morphologic criteria should be classed into biological species renewedly. Results of interspecific hybridization among seven barrenwort species showed only *E. myrianthum*, with lower grain set rate of crossing with other species (almost all under 30%), maybe a biological species. However, the others are not biological species for there are high grain set rate each other. There will be lots of works to do in studying biological species of this genus.

### 4.3. Origin of This Genus

The distribution pattern of *Epimedium* is the typical type of old world temperate zone [6], internationally distributing in three discontinuous areas of the Mediterranean Sea and Western Asia, China, and Japan. China is the diversity centre of this genus with about 50 species and five infraspecies on record [25], mostly occurring in the south-western China. However, studies on the origin of this genus still have not sure conclusions.

This study on POD isozyme showed that the means of *d*  values of the two species of the small-flowers group, *E. sagittatum *and *E. myrianthum*, were 0.432 and 0.418, respectively, lower than the large-flowers group species, implying that the small-flowers group is older in genus *Epimedium*. It is possible that the course of evolution of *Epimedium *was from the small-flowers group to the large-flowers group. Since it is known that south-western China is the origin centre of the small-flowers group of this genus [6], south-western China may also be the origin centre of the genus *Epimedium*.

## Figures and Tables

**Table 1:** *Epimedium* accessions used in this study.

Accessions	Species	Native to	Symbols	Speciman no.	Size of flower
EP04100701AC	*E. acuminatum* Franch	Guizhou, Guiyang, China	AC1	04100701	Large
EP05050709AC	*E. acuminatum* Franch	Guizhou, Yanhe, China	AC2	05050709	Large
EP05101001AC	*E. acuminatum* Franch	Guizhou, Mentan, China	AC3	05101001	Large
EP05101002AC	*E. acuminatum* Franch	Yunnan, Kunming, China	AC4	05101002	Large
EP05050801AC	*E. acuminatum* Franch	Guizhou, Dejiang, China	AC5	05050801	Large
EP05021002YI	*E. yinjiangense* M. Y. Sheng and Q. F. Chen	Guizhou, Yinjiang, China	YI1	05021002	Large
EP05021003YI	*E. yinjiangense* M. Y. Sheng and Q. F. Chen	Guizhou, Yinjiang, China	YI2	05021003	Large
EP05050403LU	*E. luodianense* M. Y. Sheng and Q. F. Chen	Guizhou, Luodian, China	LU1	05050403	Large
EP04120001LU	*E. luodianense* M. Y. Sheng and Q. F. Chen	Guizhou, Pingtan, China	LU2	04200001	Large
EP04081205SI	*E. simplicifolium* T. S. Ying	Guizhou, Yanhe, China	SI1	04081205	Large
EP04081208SI	*E. simplicifolium* T. S. Ying	Guizhou, Yanhe, China	SI2	04081208	Large
EP06012301FR	*E. franchetii* Stearn	Guizhou, Tongren, China	FR	06012301	Large
EP04041001WU	*E. wushanense* T. S. Ying	Guizhou, Kaili, China	WU1	04041001	Large
EP05051102WU	*E. wushanense* T. S. Ying	Guizhou vivariuam, China	WU2	05051102	Large
EP05032101WU	*E. wushanense* T. S. Ying	Guizhou, Guiyang, China	WU3	05032101	Large
EP04061505LE	*E. leptorrhizum* Stearn	Guizhou, Dejiang, China	LE1	04061505	Large
EP04091201LE	*E. leptorrhizum* Stearn	Guizhou, Wuchuan, China	LE2	04091201	Large
EP04061501LE	*E. leptorrhizum* Stearn	Guizhou, Yinjiang. China	LE3	04061501	Large
EP04052201LE	*E. leptorrhizum* Stearn	Guizhou, Guiyang, China	LE4	04052201	Large
EP05100801DA	*E. davidii* Franch	Yunnan, Kunming, China	DA	05101001	Large
EP04072202AL	*E. alpinum* L.	Munich, German	AL1	04072201	Large
EP04072201AL	*E. alpinum* L.	Munich vivariuam, German	AL2	04072201	Large
EP05092302PU	*E. pubigerum* (DC.) Morren and Decne.	Munich, German	PU1	05092302	Large
EP05092301PU	*E. pubigerum* (DC.) Morren and Decne.	Munich vivariuam, German	PU2	05092301	Large
EP03100202SA	*E. sagittatum* (Sieb. et. Zucc.) Maxim.	Guizhou vivarium, China	SA1	03100202	Small
EP03100201SA	*E. sagittatum* (Sieb. et. Zucc.) Maxim.	Guizhou Shibing, China	SA2	03100201	Small
EP04050401MY	*E. myrianthum* Stearn	Guizhou, Shibing, China	MY	04050401	Small

**Table 2:** Karyotype formula (KF), satellite position (SAT), total length of the haploid complement (TCL), range of chromosome length (range), intrachromosome asymmetry index (*A*_1_), interchromosome asymmetry (*A*_2_), mean centromeric index (CI), and Stebbin's asymmetry categories (ST) of the studied *Epimedium* species.

Species	KF	SAT	TCL ± SE	Range	*A*_1_	*A*_2_	CI ± SE	ST
*E. acuminatum *	4m + 6sm + 2m (SAT)	1s	45.23 ± 0.78	3.08~4.77	0.38	0.17	37.54 ± 0.15	2A
*E. yinjiangense *	4m + 6sm + 2m (SAT)*	1s	29.23 ± 2.36	2.15~2.92	0.43	0.14	35.76 ± 0.28	2A
*E. luodianense *	4m + 6sm + 2m (SAT)*	1s	47.88 ± 1.24	2.28~4.87	0.38	0.17	36.69 ± 0.31	2A
*E. leptorrhizum *	6m + 4sm + 2m (SAT)*	2s	46.00 ± 1.04	3.06~4.44	0.34	0.14	39.00 ± 0.14	2A
*E. simplicifoilum *	4m + 6sm + 2m (SAT)*	2s	45.43 ± 2.01	3.05~4.17	0.42	0.11	36.06 ± 0.52	2A
*E. wushanense *	4m + 6sm + 2m (SAT)*	2s	46.86 ± 0.88	3.33~4.44	0.33	0.10	39.40 ± 0.46	2A
*E. alpinum *	4m + 6sm + 2m (SAT)	1s	32.96 ± 1.34	2.22~3.06	0.35	0.13	38.39 ± 0.39	2A
*E. davidii *	4m + 6sm + 2m (SAT)*	1s	47.32 ± 1.67	3.33~4.72	0.42	0.16	36.08 ± 0.22	2A
*E. pubigerum *	4m + 6sm + 2m (SAT)	1s	38.59 ± 0.96	2.50~3.61	0.32	0.14	39.88 ± 0.18	2A
*E. franchetii *	4m + 6sm + 2m (SAT)*	1s	38.04 ± 0.82	2.91~4.72	0.36	0.18	38.20 ± 0.29	2A
*E. myrianthum *	4m + 6sm + 2m (SAT)*	1s	45.45 ± 1.27	3.38~4.15	0.40	0.08	36.69 ± 0.38	2A
*E. sagittatum *	4m + 6sm + 2m (SAT)	1s	41.88 ± 1.01	3.27~4.68	0.34	0.18	38.69 ± 0.13	2A

SE: standard error, m: metacentric, sm: submetacentric, st: subteleocentric, s: short arm, and *indicates first description of species.

**Table 3:** The interspecific dissimilarity coefficient (*d*, %) of POD zymogram of eleven barrenwort species and their means.

Species	FR	PU	YI	LE	LU	SI	AC	AL	WU	MY	SA	Mean ($\overset{-}{d}$)
FR												67.8
PU	76.5											66.9
YI	100	83.3										62.1
LE	81.8	37.5	66.7									58.4
LU	75.0	86.4	33.3	71.4								57.0
SI	77.8	71.4	50.0	50.0	60.0							47.1
AC	50.0	64.7	71.4	63.6	50.0	33.3						45.3
AL	75.0	69.2	33.3	42.9	50.0	20.0	50.0					44.6
WU	45.5	62.5	66.7	60.0	42.9	50.0	27.2	42.9				44.3
MY	77.8	57.1	50.0	50.0	60.0	33.3	33.3	20.0	25.0			43.2
SA	63.6	62.5	66.7	60.0	42.9	25.0	9.1	42.9	20.0	25.0		41.8
Mean ($\overset{-}{d}$)	67.8	66.9	62.1	58.4	57.0	47.1	45.3	44.6	44.3	43.2	41.8	52.59

**Table 4:** The rate of grain set (%) of hybridization between species.

♂	♀						
	*E. myrianthum *	*E. acuminatum *	*E. wushanense *	*E. letorrhizum *	*E. luodianense *	*E. simplicifolium *	*E. yinjiangense *
*E. myrianthum *		15.38	15.83	26.97	35.82	16.85	30.09
*E. acuminatum *	29.17		49.51	24.34	47.06	73.35	41.67
*E. wushanense *	33.03	60.32		28.26	63.41	92.23	64.04
*E. letorrhizum *	28.69	29.62	42.86		92.44	87.01	76.47
*E. luodianense *	35.96	43.88	80.15	67.68		73.08	88.07
*E*. *simplicifolium *	35.05	71.43	88.78	75.00	48.20		77.01
*E*. *yinjiangense *	20.80	43.08	72.73	41.38	83.33	62.92	
